# Hippocampal and cortical tissue-specific epigenetic clocks indicate an increased epigenetic age in a mouse model for Alzheimer’s disease

**DOI:** 10.18632/aging.104056

**Published:** 2020-10-20

**Authors:** Emma Coninx, Yap Ching Chew, Xiaojing Yang, Wei Guo, Amelie Coolkens, Sarah Baatout, Lieve Moons, Mieke Verslegers, Roel Quintens

**Affiliations:** 1 Radiobiology Unit, Institute for Environment, Health and Safety, Belgian Nuclear Research Centre (SCK CEN), Mol 2400, Belgium; 2 Neural Circuit Development and Regeneration Research Group, Department of Biology, KU Leuven, Leuven 3000, Belgium; 3 Epigenetics Technologies, Zymo Research Corporation, Irvine, CA 92614, USA

**Keywords:** DNA methylation, epigenetic clock, hippocampus, cortex, Alzheimer's disease

## Abstract

Epigenetic clocks are based on age-associated changes in DNA methylation of CpG-sites, which can accurately measure chronological age in different species. Recently, several studies have indicated that the difference between chronological and epigenetic age, defined as the age acceleration, could reflect biological age indicating functional decline and age-associated diseases. In humans, an epigenetic clock associated Alzheimer’s disease (AD) pathology with an acceleration of the epigenetic age. In this study, we developed and validated two mouse brain region-specific epigenetic clocks from the C57BL/6J hippocampus and cerebral cortex. Both clocks, which could successfully estimate chronological age, were further validated in a widely used mouse model for AD, the triple transgenic AD (3xTg-AD) mouse. We observed an epigenetic age acceleration indicating an increased biological age for the 3xTg-AD mice compared to non-pathological C57BL/6J mice, which was more pronounced in the cortex as compared to the hippocampus. Genomic region enrichment analysis revealed that age-dependent CpGs were enriched in genes related to developmental, aging-related, neuronal and neurodegenerative functions. Due to the limited access of human brain tissues, these epigenetic clocks specific for mouse cortex and hippocampus might be important in further unravelling the role of epigenetic mechanisms underlying AD pathology or brain aging in general.

## INTRODUCTION

The increase in life expectancy seen in developed countries is associated with an increased prevalence of chronic diseases such as cancer, neurodegeneration and cardiovascular disease [1]. This is due to the fact that aging is associated with a progressive decline in functional capacity of various cells and organs, eventually leading to disease and death [2]. Usually, the chronological age (i.e. the number of calendar years passed after birth) will deviate from biological age, which takes all health outcomes into account and is highly variable due to many interfering factors, e.g. genetics, environment and lifestyle [3]. Hence, in order to decipher how aging acts as a risk factor for chronic diseases, there is an urgent need for accurate measures of biological age [4]. A number of age predictors have been proposed to accurately measure biological age, e.g. telomere length and transcriptomic, proteomic, metabolomic and composite predictors. However, most of these biomarkers have drawbacks including a low predictive power for health span and insufficient validation [5].

Recently, the epigenetic changes associated with aging have gained much attention in the aging biomarker research field [6]. In addition to a general loss of histones and modifications of histone marks including H3K4me3, H4K16ac and H3K56ac [7], age-related changes of the epigenome include alterations in DNA methylation patterns which typically consist of 5-methylcytosine occurring at CpG dinucleotides [8]. Overall, aging induces a genome-wide loss of DNA methylation, although it can encompass hypermethylation at specific loci [9, 10]. Age-related DNA methylation changes that are highly reproducible have led to the development of so-called “epigenetic clocks”, by selecting specific CpG-sites that display age-dependent methylation states [11]. Horvath (2013) was the first to describe a human multi-tissue age-predictor based on the methylation states of 353 age-related CpGs [12]. A few years after the development of Horvath’s human epigenetic clock, a multi-tissue epigenetic age predictor in mice was published containing 329 age-associated CpG-sites [13] different from the ones selected by Horvath. These first epigenetic clocks were developed to accurately measure chronological age. Later, these and other epigenetic clocks were used to anticipate aging-related health outcomes both in humans [14–16] and in mice [17–19]. The difference between epigenetic age and chronological age, defined as the age acceleration, is already associated with mortality [20] and age-related diseases [14, 21]. Therefore, epigenetic clocks are currently considered the most promising predictor of biological age in comparison to other candidates [5, 22]. Furthermore, epigenetic clocks can be applied in developing longevity and rejuvenating interventions and in determining the impact of stress factors on biological age [23]. Although many research groups focused on multi-tissue epigenetic clocks, more accurate clocks could be established by using the methylation levels of tissue-specific CpG-sites [24], in part because differences in DNA methylation patterns are in general more pronounced between tissues than ages [25]. This may explain the sub-optimal performance of multi-tissue epigenetic clocks for biological estimation of specific tissues [26]. Therefore, tissue-specific clocks need to be developed which measure biological age in relation to age-related diseases, with the potential to serve as a prognostic and diagnostic marker of a certain age-associated disease, and to provide more insights in the underlying mechanisms involved [27–29]. Due to limited access of human tissue samples, mouse models will be of particular relevance to achieve this goal. Until now, tissue-specific clocks in mice have only been developed for blood [18, 30], liver [19] and muscle [31].

One of the most prevalent neurodegenerative diseases is Alzheimer’s disease (AD), accounting for 60-70% of the 50 million dementia patients worldwide [32]. An epigenetic clock was tested in AD patients in which an acceleration of the epigenetic age in the prefrontal cortex was suggested to be associated with the decline in cognitive function [33]. Besides the cortex, the hippocampus is also highly affected by AD contributing to the associated cognitive decline [34]. Furthermore, several AD risk factors, e.g. body mass index, cholesterol levels, blood pressure and smoking, were demonstrated to accelerate epigenetic age [35]. Further research is needed to increase our knowledge on how epigenetic age is associated with AD, and to potentially give further insights in the AD pathology. For this, animal experiments supplementing human research are very useful and enable to examine how soon during the disease process epigenetic age is modified, to determine risk factors for the accelerated aging linked to AD, and to test the efficacy of potential interventions delaying the increase in epigenetic age.

In this study, epigenetic clocks were developed and validated for mouse hippocampus and cortex. We measured the epigenetic age of cortex and hippocampus from young adult (3 months) to aged (15 months) female C57BL/6J and triple transgenic AD (3xTg-AD) mice (further referred to as B6 and AD mice, respectively), which are extensively used in AD research. The epigenetic age of AD cortex and hippocampus was found to be increased in comparison to that of B6 mice. The epigenetic age acceleration, which is thought to be associated with biological age, was elevated for the cortex in relation to the hippocampus in both mouse strains. Furthermore, the AD mice were epigenetically older when the first signs of AD pathology appeared, with slower rates of acceleration over time. Differences in DNA methylation of age-associated CpGs were more evident between brain regions than mouse strains and thirdly between various ages. Notably, several CpGs that showed opposite age-dependent DNA methylation profiles for both strains were related to neurodegenerative disease, such as AD. In addition, the most-significant age-associated CpG-sites clustered together in genomic regions encoding for developmental, aging-related and neuronal functions. Altogether, the developed brain region-specific epigenetic clocks can successfully be implemented in future research to measure brain epigenetic age to further unravel the role of epigenetics in neurodegenerative pathology.

## RESULTS

### Epigenetic clocks specific for mouse hippocampal and cortical tissue

**Figure 1:**
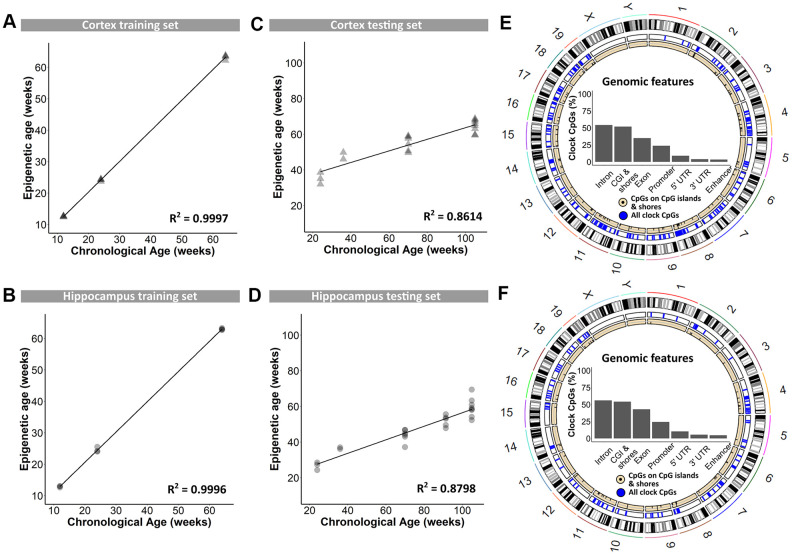
**Training and testing of the DNAge® algorithm to predict chronological age in mouse cortical and hippocampal tissue.** (**A**, **B**) A tissue-specific algorithm was designed for cortex (**A**) and hippocampus (**B**) to accurately predict chronological age. N = 3 – 4. (**C**, **D**) The DNAge® algorithm was tested on an independent sample set of male B6 mice for both cortex (**C**) and hippocampus (**D**). N = 2 – 8. (**E**, **F**) Circos plots indicate the genomic locations of clock CpG-sites in CpG-islands (black) and all clock CpG-sites (blue) in cortex (**E**) and hippocampus (**F**). Insets indicate relative distributions of clock CpG-sites across genomic features.

In a first instance, blood DNA samples were used for genome-wide reduced representation bisulfite sequencing using the Methyl-MidiSeq method from Zymo Research. This method covers ~30% of the entire methylome at single base resolution. From this, CpGs in differentially methylated regions (DMRs) were identified, and supplemented with age-dependent DNA methylation sites retrieved from the literature [13, 17, 19, 36, 37]. This generated a list of 2,031 individual CpGs in the mouse genome that were further analyzed using targeted bisulfite sequencing and used to train the proprietary DNAge® algorithm (see methods). DNA methylation levels of cerebral cortex and hippocampus, dissected from B6 mice at 12, 24 and 64 weeks of age, served to train the algorithm. Separate algorithms were built specifically for cortex and hippocampus using respectively 1,144 and 732 CpGs out of the original 2,031 CpGs with 436 overlapping loci (see Supplementary Table 1). Both algorithms could very accurately predict epigenetic age as observed by almost perfect correlation to the chronological age of 0.9997 for cortex and of 0.9996 for hippocampus (Figure 1A and B). Both the cortex- and hippocampus-specific epigenetic clocks were subsequently validated on an independent sample set from 24-, 36-, 70-, 91- and 105-week old male B6 mice. Also in this validation phase, the cortical and hippocampal clocks performed very well, with coefficients of determination of 0.8614 and 0.8798, respectively (Figure 1C, 1D). The distribution of clock CpG-sites across genomic features was very comparable between the cortex and hippocampus, with a majority of them found in introns and CpG-islands (Figure 1E, 1F), of which the latter are inherently enriched in CpGs [38].

### AD mice show an increased epigenetic age compared to B6 mice

**Figure 2:**
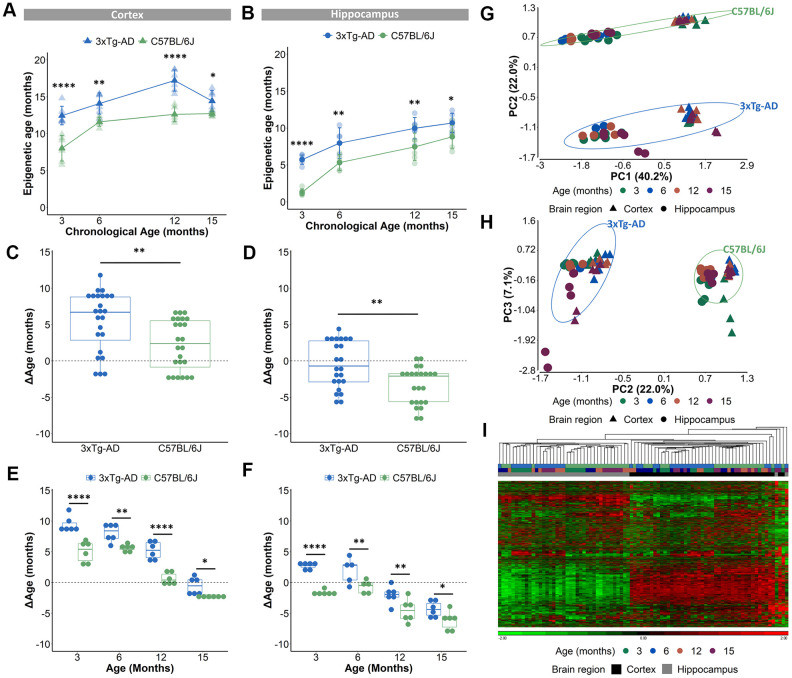
**Acceleration of epigenetic age in AD mice compared to B6 mice in cortical and hippocampal tissue.** (**A**, **B**) The predicted epigenetic age for cortex (**A**) and hippocampus (**B**) of AD (blue) and B6 (green) mice. Mean ± SD. (**C**, **D**) The mean ΔAge and (**E**, **F**) the ΔAge per chronological time point for cortex and hippocampus of AD (blue) and B6 (green) mice. N = 6. * p < 0.05, ** p < 0.01, **** p < 0.0001. (**G**, **H**) A principal component analysis (PCA) with PC1 and PC2 (**G**) and PC2 and PC3 (**H**) based on the DNA methylation value of 1696 CpGs in AD (blue) and B6 (green) mouse cortex (triangles) and hippocampus (circles). Age is colored by month. (**I**) Based on the same 1696 CpGs, unsupervised hierarchical clustering was performed using the AD (blue) and B6 (green) mouse cortex (black) and hippocampus (grey).

The validated epigenetic clocks were then used to estimate the epigenetic ages of cortical and hippocampal tissues from 3-, 6-, 12- and 15-months old female B6 and AD mice (the DNA methylation values of the 2,031 CpG-sites can be found in Supplementary Table 2). Only female mice were used for this experiment as male AD mice show a more subtle AD pathology compared to female mice, which was obvious in our colony [39] and also reported by different research groups [40, 41]. Based on the DNAge® algorithms for cortex and hippocampus, the predicted epigenetic age of AD mice was significantly augmented in both cortex and hippocampus for all ages (Figure 2A, 2B). ΔAge which compared epigenetic age to chronological age was used to measure a potential acceleration or deceleration of aging. The ΔAge was higher in cortical tissue, compared to hippocampal tissue suggestive for a specific epigenetic aging speed for these distinct brain regions (Supplementary Figure 1A, 1B). We determined that the AD cortex was 6.7 months older (median difference) compared to their chronological age, while the hippocampus was 0.7 months younger (Figure 2C, 2D). As the mean lifespan of female AD mice is 17 months [42], we indicated for the cortex a 39.4% acceleration of their lifespan and for the hippocampus a 4.1% deceleration. Compared to their chronological age, the B6 cortex was 2.4 months older, while the hippocampus was 2.1 months younger (Figure 2C, 2D). Taking into account that the mean lifespan of female B6 mice is 25 months [43], the epigenetic clock revealed a 8.4% lifespan acceleration for the cortex and a 9.6% lifespan deceleration for the hippocampus. To reveal a potential increased age acceleration in the pathological mouse model, the ΔAge of B6 mice was compared to that of AD mice. This disclosed that, based on the DNAge® algorithm trained in B6 mice, the AD cortex had a positive age acceleration compared to the B6 cortex with a median difference of 4.3 months, while the median difference of the hippocampus was 1.4 months (Figure 2C, 2D). In line with previous studies [27], we observed non-linear epigenetic age trajectories, with slower rates of epigenetic aging over time. The epigenetic age of the B6 cortex accelerated (positive ΔAge) until 12 months of age, while for AD mice an acceleration was visible until 15 months of age (Figure 2E and Supplementary Figure 1C, 1D). For hippocampal tissue, the epigenetic age of B6 mice decelerated (negative ΔAge) at all time points. This is in contrast to the AD mice, in which epigenetic age was first accelerated at 3 and 6 months of age and then decelerated at 12 and 15 months of age (Figure 2F and Supplementary Figure 1C, 1D).

**Figure 3:**
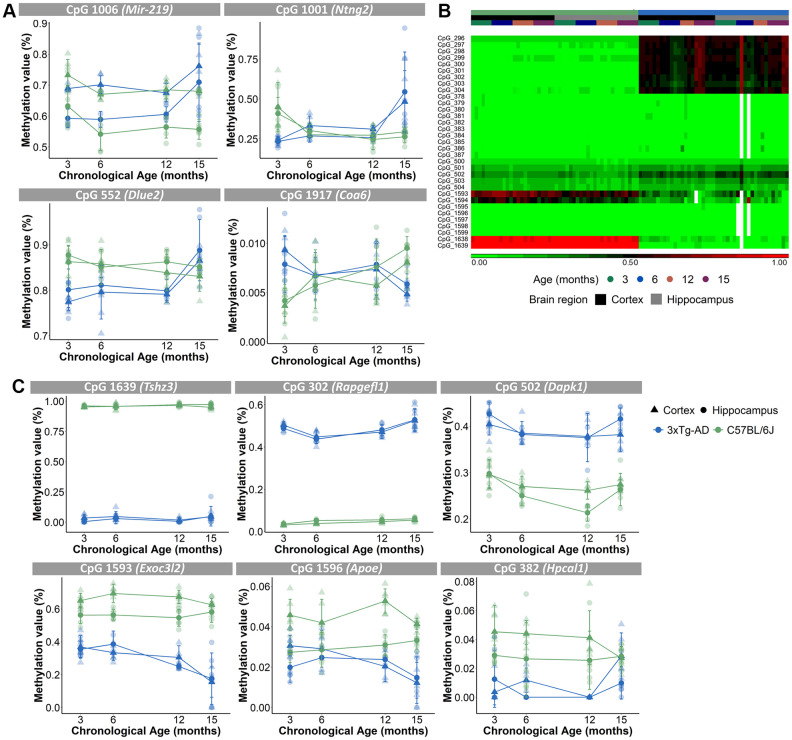
**Different age-associated methylation profiles for AD mice compared to B6 mice.** (**A**) Methylation profiles of CpGs in close proximity of *Mir-219*, *Ntng2*, *Dlue2* and *Coa6* genes for the cortex (triangles) and hippocampus (circles) were opposite for the AD (blue) and B6 (green) mice. N = 4. Mean ± SD. (**B**) Unsupervised hierarchical clustering using AD (blue) and B6 (green) mouse cortex (black) and hippocampus (grey) for 33 of the 157 CpG-sites that were differentially methylated for strain. (**C**) Methylation profiles for the cortex (triangles) and hippocampus (circles) of CpGs, associated with AD-related genes *Tshz3, Rapgefl1, Dapk1, Exoc3l2, Apoe and Hpcal1* genes, show differing values between AD (blue) and B6 (green) mice.

Because a substantial number of the 2,031 CpG loci had missing DNA methylation values, we decided to exclude those CpGs for further analyses, which resulted in 1,696 CpGs (see Methods). A principal component analysis (PCA), executed to analyze the variation within the methylation values of the 1,696 CpGs, showed that samples were mostly separated by brain region (PC1), then by mouse strain (PC2) and only thirdly by age (PC3) (Figure 2G, 2H). Performing an unsupervised hierarchical clustering based on the methylation values of the 1,696 CpG-sites, similarly revealed a clear separation of the samples primarily based on brain region (Figure 2I). Thus, our analysis showed that despite the existence of overlapping CpG loci, the cortex and hippocampus display very different levels of DNA methylation of age-associated CpGs. Furthermore, based on a 3-way ANOVA analysis on the 1,696 CpGs we identified 59 CpGs that showed significantly (*p*-value <10E-5 for the interaction between strain and age) different age-associated methylation profiles between the mouse strains. These CpGs were found in clusters in the proximity of certain genes, namely 7 CpGs in *Mir-219*, 6 CpGs in *Ntng2*, 5 CpGs in *Dlue2* and 2 CpGs in *Coa6.* For *Mir-219*, *Ntng2* and *Dlue2* methylation levels increased with age for the AD mice and decreased with age for the B6 mice. This is in contrast to *Coa6*, for which methylation levels decreased for the AD mice and increased for the B6 mice (Figure 3A). Finally, 157 CpGs were identified with different DNA methylation values (ANOVA *p*-value <10E-5 for strain) between AD and B6 mice (Figure 3B). Several of these CpG loci clustered in or near genes (*Tshz3*, *Rapgefl1*, *Exoc3l2*, *Dapk1*, *Apoe* and *Hpcal1*) that have been linked to AD in humans and/or mice (Figure 3C).

### The most significant age-associated CpGs cluster together in genomic regions important for developmental, aging-related and neuronal functions

**Figure 4:**
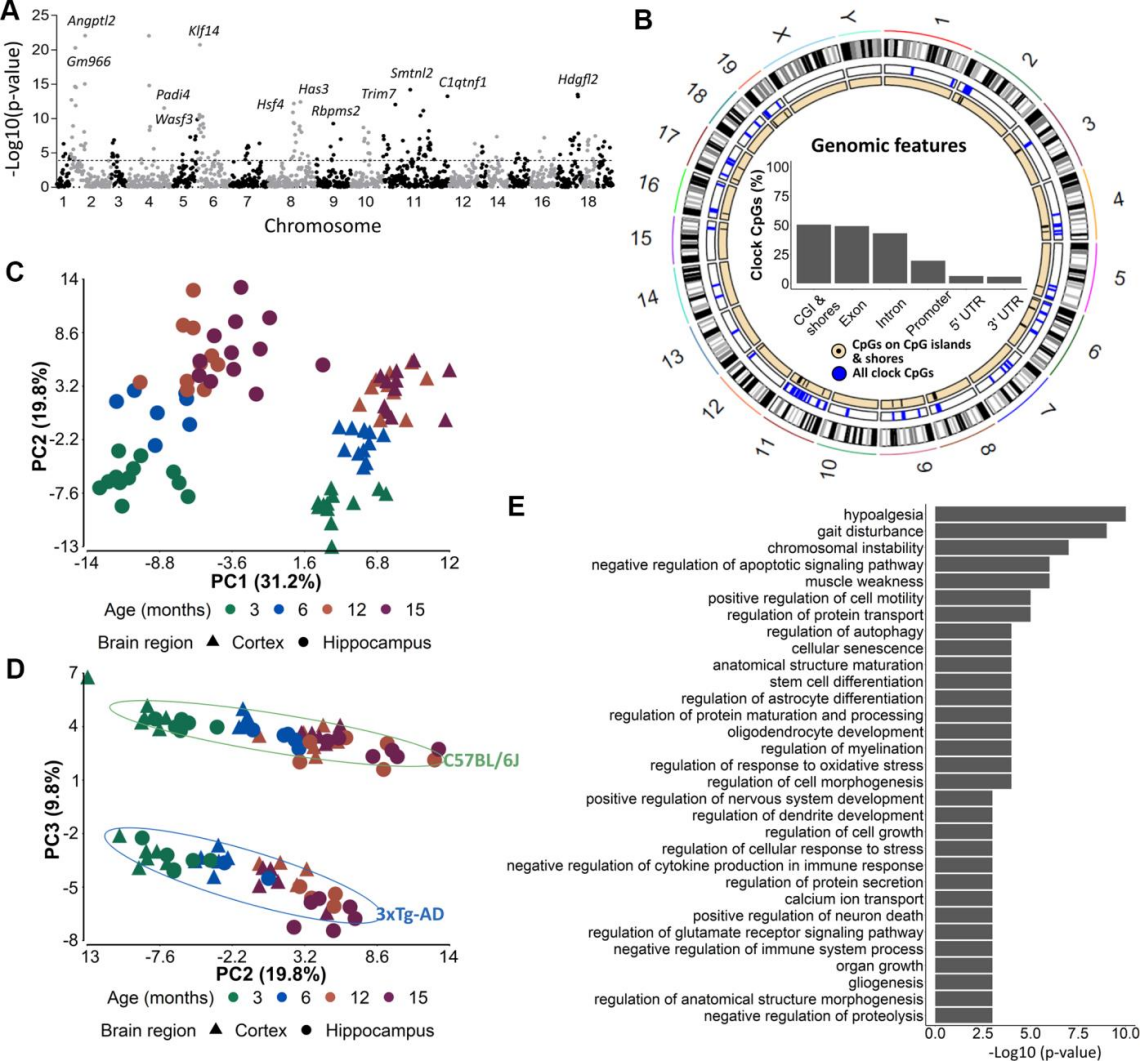
**The 175 most significant age-associated CpGs cluster together in genomic regions important for developmental, aging-related and neuronal functions.** (**A**) A Manhattan plot of the 1696 CpG-sites with 10E-4 as cut-off value indicating the 175 most significant age-associated CpGs. (**B**) Circos plots of genomic locations of the 175 CpGs in CpG-islands (black), and all clock CpG-sites (blue). Insets indicate relative distributions of clock CpG-sites across genomic features. (C, D) A principal component analysis (PCA) with (**C**) PC1 and PC2 and (**D**) PC2 and PC3 based on the DNA methylation value of 175 most significant age-associated CpGs in AD (blue) and B6 (green) mouse cortex (triangles) and hippocampus (circles). Age is colored by month. N = 6. (**E**) The GREAT analysis associated the 175 CpGs with a regulatory domain in the mouse genome.

In order to identify the CpGs that were mostly associated with age, we performed a 3-way ANOVA analysis on the 1,696 CpG-sites with brain region, mouse strain and age, as factors. To further investigate the most differentially methylated CpGs according to age, we selected 175 CpGs out of the 1,696 CpG-sites with a *p*-value for age <10E-4 (Figure 4A). Concerning the distribution of these CpGs among genomic features, we observed a slight shift towards more exon- and fewer intron-related CpGs (Figure 4B) as compared to the original 2,031 CpG-sites. A PCA performed on the 175 CpGs still showed a clear distinction of brain region (PC1) and strain (PC3) (Figure 4C). In addition, samples could now also be separated based on age (PC2) (Figure 4D) for both strains.

We used the genomic regions enrichment of annotation tool (GREAT) [44] to assign biological meaning to the 175 most important age-associated CpG-sites that were related to 128 genes (Supplementary Table 3). Some of the genes were related to aging, e.g. *Hsf4* and *Klf14*, to developmental processes, e.g. *Tbx2* and *Dmbx1*, to synaptic plasticity, e.g. *Srcin1*, *Calb2* and *Neurl1a* and to AD, e.g. *Bin1*. Among the biological processes that were enriched we found processes related to development (e.g. nervous system development, organ growth, gliogenesis), aging and age-related syndromes (e.g. hypoalgesia, gait disturbance, muscle weakness and cellular senescence), and neuronal/brain function (astrocyte differentiation, protein transport and secretion, dendrite development, glutamate receptor signaling, calcium ion transport) (Figure 4E). By using the UCSC genome brower’s LiftOver program, we could successfully convert 152 out of the 175 most significant age-associated CpG loci from the mouse (mm10) to human (hg19) genome assemblies [45], indicating high conservation of the loci. However, no overlap was found with the 513 CpG loci used in the phenoAge clock developed by Levine et al. used to correlate cortical epigenetic age and AD-related cognitive decline in humans [14].

## DISCUSSION

Compared to multi-tissue epigenetic clocks, tissue-specific clocks may be more suitable as indicators of disease-associated aging and may provide deeper insight into pathophysiological mechanisms, especially in cases where certain tissues are particularly affected [27]. One such disease is AD, an age-associated disease characterized by intracellular neurofibrillary tangles and the accumulation of amyloid-β plaques with aging as the major risk factor [46]. However, for obvious reasons it is not always possible to obtain tissue samples from humans. Therefore, we have generated in this study epigenetic clocks from cortex and hippocampus of a wild-type and an AD mouse model. To generate these clocks, we measured DNA methylation values of 2,031 CpGs using targeted bisulfite sequencing allowing 1000x coverage, which is several orders of magnitude higher than that of other studies [13, 18, 19]. We should acknowledge that even though we reached high coverage, missing values are still an inherent issue. However, as aging-related changes are pervasive in the DNA methylome [24], we are confident to have accurately captured the age-related variations in methylation values at the investigated CpGs. More than 100x coverage is required to obtain high-confidence differentially methylated positions [47].

The clocks we eventually developed consisted of 1, 144 and 732 CpGs for cortex and hippocampus, respectively, of which 436 overlapped. Despite this high overlap, both the methylation values of several individual CpGs as well as the epigenetic age predictions were very different between cortex and hippocampus. These differences may at least partly be explained by cell type heterogeneity (neuronal and non-neuronal) between both brain regions which are mostly marked by different pyramidal neuron subtypes and different ratios of mural, endothelial and glial cells [48, 49]. It can furthermore be anticipated that this heterogeneity is also influenced by changes in cellular composition during aging. For instance, the rates of adult neurogenesis and gliogenesis are higher in the hippocampus compared to the cortex [50] and may therefore result in a relative increase of biologically “younger” cells. This may contribute to our finding that the biological age of the hippocampus was lower than that of the cortex. However, although cell type-specific differences in DNA methylation patterns have recently been identified in the human and mouse frontal cortex [51], the genome-wide methylation patterns between neuronal and especially non-neuronal cells in the cortex and hippocampus were found to be very comparable in human post-mortem cortex and hippocampus [52]. We hypothesize that the observed distinct epigenetic age and age acceleration predictions may reflect enhanced biological aging of the cortex compared to the hippocampus in mice. This seems to be different in humans, where no difference in epigenetic age acceleration could be observed between different cortical regions and the hippocampus [53]. The reason for this discrepancy may be the selection of CpGs used to train the clocks. For the human study the Horvath clock was used [12] which was originally designed to perfectly predict chronological age.

The development of these epigenetic clocks specific for mouse cortex and hippocampus that were built from samples from a rather broad range of ages is important. Existing DNA clocks have been shown to work in general relatively well across different tissues, but underestimation of epigenetic age in older samples was particularly observed in brain [54]. A recently developed human cortical-specific DNA methylation clock outperformed the predictive accuracy for cortical age estimation in comparison with existing clocks designed for different tissues [55]. This indicates the need for tissue-specific epigenetic clocks, which may also better reflect tissue-specific changes in DNA methylation due to biological aging and therefore more accurately predict tissue-specific aging-related diseases.

Although both our clocks are able to estimate chronological age, they are not perfect. This is in fact essential in order to allow their use in predicting biological age, because by definition perfect chronological clocks cannot contain information on variation in biological age [56]. Indeed, there should be room for variability in order to generate biological aging associations [57]. It was proposed that epigenetic clocks specific for biological aging should focus on a defined aspect of the aging biology, including disease-related factors [27]. Here, we used AD mice to compare epigenetic aging of the cortex and hippocampus to that of non-pathological B6 mice over an extended period of their life. We found accelerated epigenetic aging for both brain regions in the AD mice, especially during early life, when also DNA methylation changes are most dynamic [10, 58]. It has been proposed that early-life changes in DNA methylation patterns may direct gene expression related to aging and age-related diseases later in life [59]. Therefore, the difference in DNA methylation observed at 3 months of age in the AD mice, which resulted in an increased estimated epigenetic age, could eventually participate to the increased aging phenotype observed with AD. In this respect, it is important to note that already at the age of 3 months, AD mice have started to develop aspects of the AD pathology. The presence of Aβ oligomers in the cortex can therefore potentially be related to the increase in epigenetic age. DNA methylation changes at certain CpG-sites were suggested to be linked to AD pathology [60]. As some of these epigenetic changes could be detected in presymptomatic subjects, it was proposed that they may have a role in the onset of AD [60].

Another possible explanation of this apparent epigenetic age acceleration of AD mice may be the difference in genetic background between the AD and B6 mice. However, previous research already demonstrated no epigenetic age acceleration when comparing different mouse strains [18, 19]. Moreover, this would not necessarily explain the differences we observed in age-associated DNA methylation between specific CpGs. Importantly, among the relatively few genes that showed opposite age-dependent DNA methylation profiles, several have shown to be related to neurodegenerative diseases and AD. For instance, we observed 7 CpGs in *Mir-219* displaying an overall reduction in methylation with age in B6 mice, while being increased in AD mice. *Mir-219* expression is known to be reduced in brains of AD patients [61], and suggested to promote neurodegeneration and disease progression [62]. A similar age-dependent methylation profile was observed for 5 CpGs in *Dleu2*. Also this gene is downregulated in the brain of human AD subjects [63]. CpGs near other AD-related genes, like *Tshz3* [64], *Rapgefl1* [65, 66], *Exoc3l2* [67, 68], *Dapk1* [69], *Apoe* [70] and *Hpcal1* [71] were differentially methylated between B6 and AD mice. Furthermore, Other CpG loci contributing to our cortical and hippocampal clocks are proximal to the mouse orthologues of genes found to be differentially methylated in AD patients, including *Bin1*, *Ezh1*, *Irx3*, *Rufy4*, *Dleu2* and *Ddr1* [60, 72–74], indicating the translational potential of some of these epigenetic marks. Whether the differences in DNA methylation of these genes underlie variations in their expression that may subsequently affect AD pathology or neurodegeneration in the AD model needs to be further investigated.

Several of the CpGs we identified as being the most significantly differentially methylated with age were found to be associated with genes that either play a role in important neurological functions or are known to be related to neurodegeneration and AD. *Hdgfl2*, expressed by neurons, astrocytes and oligodendrocytes in adult mouse brain tissue, has a role in cell proliferation and cell survival [75]. Both *Calb2* and *Srcin*1 function in synaptic plasticity [76–78]. Finally, *Bin1* is identified as the second most important risk factor for late onset AD after *ApoE*, which itself contains 5 CpGs among the 2,031 used for the development of our clock. *Bin1* modulates tau pathology and affects cellular functions like endocytosis, inflammation, calcium homeostasis and apoptosis [79, 80]. Although it is well known that DNA methylation in promoter regions influences gene expression [81], the link between age-dependent changes in methylation and gene expression is still unclear. For some genes a correlation has been identified, but this cannot be generalized [82]. Like DNA methylation, also gene expression is mostly regulated during the initial stages of postnatal life [83]. Therefore, it would be important to investigate a possible correlation between the transcriptome and the epigenome especially in young subjects.

We provide here the first epigenetic clocks for two mouse brain regions, based on DNA methylation data from young to old mice, and validated them using a healthy and pathological mouse strain. We believe that these clocks will be very useful for research on different neurological and neurodegenerative disorders that are, at least partly, initiated by epigenetic mechanisms.

## MATERIALS AND METHODS

### Workflow

Zymo Research designed a DNAge® mouse epigenetic aging clock targeting 2,031 age-associated CpG loci. These epigenomic loci were identified using genome-wide reduced representation bisulfite sequencing (RRBS) using the Methyl-Midiseq method to select the most differentially methylated regions (DMRs) in blood samples from 12-, 24- and 64-week old male B6 mice. Blood has been proposed to be a good surrogate to study age-dependent DNA methylation profiles in the brain [84]. These in-house detected CpGs were furthermore supplemented with age-related CpG loci found after an extensive literature search [13, 17, 19, 36, 37]. The epigenetic clock was trained on two sample sets specific for hippocampus and cortex of the same male B6 mice from 12, 24 and 64 weeks of age. In the training phase, only male mice were used to exclude gender- and hormonal differences. Afterwards, the hippocampal and cortical epigenetic clocks were tested on a separate pool of hippocampal and cortical samples from male B6 mice of 24, 36, 70, 91 and 105 weeks of age. Finally, both clocks were used to determine biological age of female B6 and AD mice of 3, 6, 12 and 15 months of age.

### Animals

The B6 mice were obtained from Janvier (Uden, The Netherlands). The AD mice contain two human transgenes, i.e. amyloid precursor protein (APPSwe) and microtubule-associated protein tau (tauP30IL), on a presenilin (PS1M146V) knock-in mice with a mixed B6;129/SvJ background, and were purchased from the Jackson Laboratory (California, USA). Full pathology of the AD mice is described by Oddo et al. [85] and revised by Belfiore et al. [86]. Female mice were bred and maintained in the animal facility of the nuclear research center SCK CEN under Specific pathogen Free (SPF) conditions and 12h light-dark cycle. Food (Altromin 1324, Carfill) and water were provided *ad libitum*. All animal experiments were performed in agreement with the Belgian laboratory animal legislation and the European Communities Council Directive (2010/63/EU) and approved by the Ethical Committee Animal Studies of the Medanex Clinic (EC_MxCl 2018-105).

### Sample collection and processing

For the training and testing samples, cortex and hippocampus were collected from male B6 mice at different ages and stabilized for downstream processing using the DNA/RNA Shield™ reagent (Zymo Research). Genomic DNA was purified using the Quick-DNA™ Miniprep Plus kit (Cat. No. D4068). For the experimental samples, cortex and hippocampus were collected from female B6 and AD mice at different ages and immediately snap-frozen in liquid nitrogen. From the cortex and hippocampus of one brain hemisphere, genomic DNA was isolated using the QIAamp DNA mini kit (Qiagen). The genomic DNA was further processed to quantify DNAge®.

### DNA methylation pre-processing

Sample library preparation and data analysis for mouse DNAge® were performed by the service provider (Zymo Research). Two-hundred ng of genomic DNA was bisulfite converted using EZ DNA Methylation-Lightning™ Kit (Zymo Research; Cat. No. D5030). Bisulfite-converted DNA libraries for targeted bisulfite sequencing platform, called SWARM® (Simplified Whole-panel Amplification Reaction Method) was prepared according the to the manufacturer’s instructions and were sequenced on a HiSeq 1500 sequencer for >1,000X coverage. Sequence reads were identified using Illumina base-calling software and aligned to the reference genome using Bismark [87–90], an aligner optimized for bisulfite sequence data and methylation calling. The methylation level of each sampled cytosine (DNA methylation value) was estimated as the number of reads reporting a C, divided by the total number of reads reporting a C or T. Thus, DNA methylation values range from 0 (completely un-methylated) to 1 (completely methylated).

### DNAge® prediction

DNA methylation values of 2,031 age-related CpG loci were used for epigenetic age prediction using Zymo Research’s proprietary mouse DNAge® algorithms: A penalized regression model’s coefficients b_0_, b_1_, ..., b_n_ related to transformed age as in equation (1):

(1) F(chronological age) = b_0_ + b_1_CpG_1_+ ⋯ + b_n_CpG_n_ + error;

DNAge® was estimated as in equation (2):

(2) DNAge® = inverse.F(b_0_ + b_1_CpG_1_+ ⋯ +b_n_CpG_n_)

The ΔAge was calculated as in equation (3):

(3) ΔAge = DNAge® – chronological age.

### Further analysis of age-associated CpGs

To obtain the distribution of the clock CpG-sites across genomic features, the annotatr R package was used at https://bioconductor.org/packages/release/bioc/vignettes/annotatr/inst/doc/annotatr-vignette.html. This package includes following CpG and genic annotations: CpG islands are the basis for all CpG annotations, and are given by the AnnotationHub package. CpG shores are defined as 2 kb upstream/downstream from the ends of the CpG islands, less the CpG islands, promoters defined as <1 kb upstream of the TSS, 5’UTR, exons, introns, coding sequence (CDS) of gene, 3’UTR and intergenic region. Enhancers were defined according to FANTOM5. Among the 2,031 CpG-sites some had missing values because of undetectable CpG loci due to failed enrichment of the amplicon or a lower DNA quality or DNA integrity. After discarding CpGs with missing values 1,696 CpG-sites remained for further analysis. From these, the 175 most significant age-associated CpG-sites were identified via 3-way ANOVA (p-value(age) <10E-4). These were used to identify enriched pathways using GREAT analysis [44] with default settings (Proximal: 5.0 kb upstream, 1.0 kb downstream, plus Distal: up to 1000 kb) and the 2,031 CpG sites as background regions. CpGs with a p-value <10E-5 for the interaction between age and strain, were considered to have significantly different age-dependent DNA methylation profiles between B6 and AD mice.

### Statistical analysis

The predicted epigenetic ages of the B6 and AD mice, estimated by the epigenetic clocks specific for hippocampus and cortex were analyzed with linear-mixed models just like the resulting ΔAge. Only for the comparison of the mean ΔAge an unpaired student T-test was performed. The statistical outcomes of the linear-mixed models for Figure 2 and Supplementary Figure 1 are enclosed in Supplementary Table 4. All statistical analyses were performed in the R statistical environment (Version 3.6.1).

## Supplementary Material

Supplementary Figure 1

Supplementary Table 1

Supplementary Table 2

Supplementary Table 3

Supplementary Table 4
