# Supplementary figures and images for: Hippocampal and cortical tissue-specific epigenetic clocks indicate an increased epigenetic age in a mouse model for Alzheimer’s disease
Source: Aging (Albany NY). 2020 Oct 20;12(20):20817–34. doi: 10.18632/aging.104056 (PMC7655172; doi:10.18632/aging.104056)

[www.aging-us.com](http://www.aging-us.com)

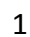

Supplement: Supplementary Figure 1 [file aging-12-104056-s001..pdf]
